# Trends of Polychlorinated Compounds in the Surroundings of a Municipal Solid Waste Incinerator in Mataró (Catalonia, Spain): Assessing Health Risks

**DOI:** 10.3390/toxics8040111

**Published:** 2020-11-22

**Authors:** Martí Nadal, Montse Marquès, Montse Mari, Joaquim Rovira, José L. Domingo

**Affiliations:** 1 Laboratory of Toxicology and Environmental Health, School of Medicine, IISPV, Universitat Rovira i Virgili, Reus, 43201 Catalonia, Spain; montserrat.marques@urv.cat (M.M.); joseluis.domingo@urv.cat (J.L.D.); 2 Environmental Engineering Laboratory, Departament d’Enginyeria Quimica, Universitat Rovira i Virgili, Tarragona, 43007 Catalonia, Spain; montserrat.mari@urv.cat (M.M.); joaquim.rovira@urv.cat (J.R.)

**Keywords:** environmental pollution, human exposure, health risks, municipal solid waste incinerator, Mataró (Catalonia, Spain)

## Abstract

Since 2008, the environmental levels of polychlorinated compounds near a municipal solid waste incinerator in Mataró (Catalonia, Spain) have been periodically monitored. The present study aimed at updating the data regarding the temporal changes occurred between 2015 and 2017, when air and soil samples were collected again, and the concentrations of the same chemical pollutants (i.e., polychlorinated dibenzo-*p*-dioxins and dibenzofurans (PCDD/Fs), and polychlorinated biphenyls (PCBs)) were analysed. Furthermore, the health risks associated with their human exposure were also evaluated. The levels of all the contaminants in soil were far below the threshold established by regional and national legislations, also being lower than those observed in previous surveys. A similar trend was also noted for PCDD/Fs in air samples, while airborne PCBs were the only group of chemicals whose levels significantly increased. In any case, the global assessment of the data regarding the different pollutants and matrices indicates that there has not been a general increase in the environmental pollution around the facility. In addition, the environmental exposure to PCDD/Fs and PCBs by the population living nearby is still clearly lower than the dietary intake of these same chemical pollutants.

## 1. Introduction

In the past, waste incineration was considered one of the most significant emission sources of a wide range of chemical pollutants [1,2]. These facilities were linked with the release of toxic and carcinogenic substances, such as heavy metals, but especially with the emission of polychlorinated dibenzo-*p*-dioxins and dibenzofurans (PCDD/Fs) [3]. However, the role of waste incineration as a contributor to the PCDD/Fs environmental burdens has progressively decreased in front of other potential emission sources (e.g., agricultural burning, cement plants, and metal production activities) [4,5,6]. The application of legislative measures, such as the EU Directive on Waste Incineration in 2000 and the Stockholm Convention in 2004, helped to reduce the contribution of waste incineration as PCDD/Fs emission sources, according to different national and international inventories [1]. Coudon et al. [7] recently published an inventory indicating that, in France, the emissions of PCDD/Fs from waste incineration decreased by 99.5% between 1990 and 2008. In addition, this sector was the main contributor, meaning 95%, 85%, and 75% of the total emissions in 1990, 2000, and 2005, respectively. However, ferrous and non-ferrous metal production facilities became the most important air releasers of PCDD/Fs between 2006 and 2008 [7]. In a flow analysis study conducted in Spain, Fuster et al. [8] found that the only municipal solid waste incinerator (MSWI) in Tarragona Province contributed to only 0.04% of the PCDD/Fs total emissions. In turn, cement kilns played a key role, with 54% of the total emissions. According to the European Dioxin Air Emission Inventory conducted in 2004, iron ore sintering was pointed out as the most important source of PCDD/Fs emissions, followed by municipal waste incineration [9]. In any case, MSWIs, medical waste incinerators and hazardous waste incinerators are still important stationary sources of PCDD/Fs, in addition to metal production processes, cement plants as well as heat and power plants [10,11].

The MSWI of Mataró (Catalonia, Spain) has been operating since 1994. The facility has undergone several legislative changes, including the EU Directives on waste incineration (2000/76/EU) and industrial emissions (2010/75/EU). In addition, the MSWI has been also conducting a large biological monitoring survey, which was initiated in 1995 [12,13]. According to the most recent data, the concentrations of PCDD/Fs and marker polychlorinated biphenyls (PCBs) in blood from people living near the facility were slightly reduced compared to the levels found in 1999 [14]. In parallel, an environmental monitoring study has been periodically carried out since 2008, providing information on the environmental burdens of PCDD/Fs and PCBs near the plant [15]. The analysis of the temporal trends of pollutant concentrations in samples of soil and air collected around the MSWI did not show any change with time [16], even after the start-up of the new Integrated Waste Recovery Centre (IWRC) of Maresme, which took place in 2010. This IWRC included the operation of a new MSWI-linked facility for the treatment of the refuse waste fraction. Summing up, important legislative and operative changes have occurred for 20 years, showing the need of a continued surveillance of potential air emissions.

The present investigation aimed at evaluating the temporal trends in the levels of PCDD/Fs and PCBs in samples of air and soil collected in the surroundings of the MSWI of Mataró. Furthermore, the health risks associated to the exposure to those pollutants were again assessed.

## 2. Materials and Methods

### 2.1. Sampling

In November of 2015 and November of 2017, two new environmental monitoring campaigns were conducted. Samplings points slightly differed from those of previous surveys [15,16]. They were relocated according to the results of the biological surveillance program [13].

**Figure 1:**
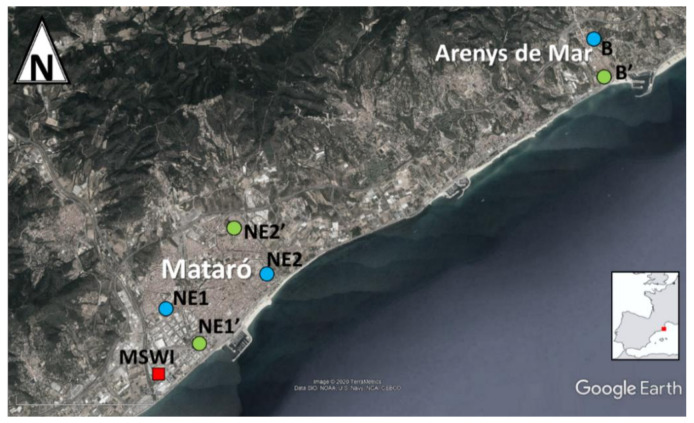
Sampling area. Blue sites indicate locations where samples of both soil and air were collected, while green sites indicate points where only soil was sampled.

Nine soil samples were obtained: three in the closest area (<25 m), two at nearby locations (<1.5 km, NE1 and NE1′), two at distant points (>3 km, NE2 and NE2′), and finally, two at Arenys de Mar (>10 km, B and B′). Five air samples were collected: two very close to the MSWI (<25 m), one in a nearby place (<1.5 km, NE1), one in a distant place (>3 km, NE2), and finally one in the town of Arenys de Mar (B). The location of the sampling points is depicted in Figure 1.

The sampling procedure was the same as that performed in previous surveys. Bulk samples of surface soils (0–5 cm of depth), consisting in five subsamples from an area of 25 m^2^, were collected. After sampling, soils were stored in polyethylene bags and dried at room temperature. Once dried, they were passed through a 2 mm sieve to homogenize the particle size. The air sampling of PCDD/Fs and PCBs was conducted by means of a TE-1000 high-volume active air sampler (Tisch Environmental, Cleves, OH, USA). The particle phase was collected on quartz microfiber filters, while the gas phase was retained in pre-cleaned polyurethane foams (PUFs). Both phases were separately stored and subsequently analysed. Air sampling for organic chemicals lasted 48 h, and an air volume of 530–607 m^3^ was obtained. PUFs and microfiber filters were stored in an airtight amber glass container with a Teflon cap to prevent the photodegradation of organic compounds.

The meteorological conditions were also monitored during the sampling. Data on temperature, atmospheric pressure, rainfall, as well as wind direction and wind speed were gathered from the Meteorological Service of Catalonia (www.meteo.cat). The average temperature during sampling was 9.2 °C, ranging between −0.3 °C and 19.9 °C. The predominant winds in the study area blow from north-northwest. The accumulated rainfall during the month prior to sampling was 77.1 mm, while during sampling no rain was recorded.

### 2.2. Analytical Determination

The procedure for the analysis of PCDD/Fs and PCBs in environmental samples has been detailed elsewhere [17,18,19]. Appropriate ^13^C_12_-labeled recovery patterns were added to the sample to check any losses during the analytical process. The extraction was performed with an ASE 300 Accelerated Solvent Extractor (Dionex Corporation, Thermo Fisher Scientific, Waltham, MA, USA), using toluene (E. Merck, Darmstadt, Germany) as solvent. The extraction operation was performed in triplicate. The subsequent clean-up consisted of a solid–liquid absorption chromatography in an open column eluted by gravity. Purification was carried out using a Merck 60 neutral column (mixture of basic, neutral, and acid activated silica gel) and an Alumina B Super 1 ICN column. Finally, the solution was brought to dryness by a stream of nitrogen, and re-dissolved with a mixture of ^13^C_12_-labeled internal standards.

The analysis of the purified extract was performed by high-resolution gas chromatography (HRGC) coupled to high-resolution mass spectrometry (HRMS). Specifically, a Fisons CE 8000 GC gas chromatograph coupled to a VG Autospec Ultima system was used for the analytical determination. A column of molten silica (30 m × 0.25 mm × 0.15 µm) was used. The detection was performed by electronic impact mass spectrometry (EI) in positive mode (35–45 eV) and with monitoring of the selected ion (SIM) with a resolution of 8000–10,000 amu. The quantification of PCDD/Fs and PCBs was performed by using internal standards. Every batch of 10 samples, a blank was analysed to detect any background contamination. Recoveries of each individual congener of PCDD/Fs ranged from 23% to 110% in soils, and from 47% to 130% in air samples.

### 2.3. Statistical Analysis

For the calculation of toxic equivalents, the World Health Organization (WHO-TEF) toxic equivalence factors were used [20]. For all those congeners with levels below their respective limit of detection (LOD), a concentration equal to one-half of this value was assumed. All data were analysed by using the statistical package SPSS 25.0. To assess the distribution of the values, the Kolmogorov-Smirnov test was used. ANOVA and subsequent T3 Dunnett’s post-hoc tests were employed to assess any significant differences in the temporal evolution. The level of statistical significance was established at *p* < 0.05.

## 3. Results

**Figure 2:**
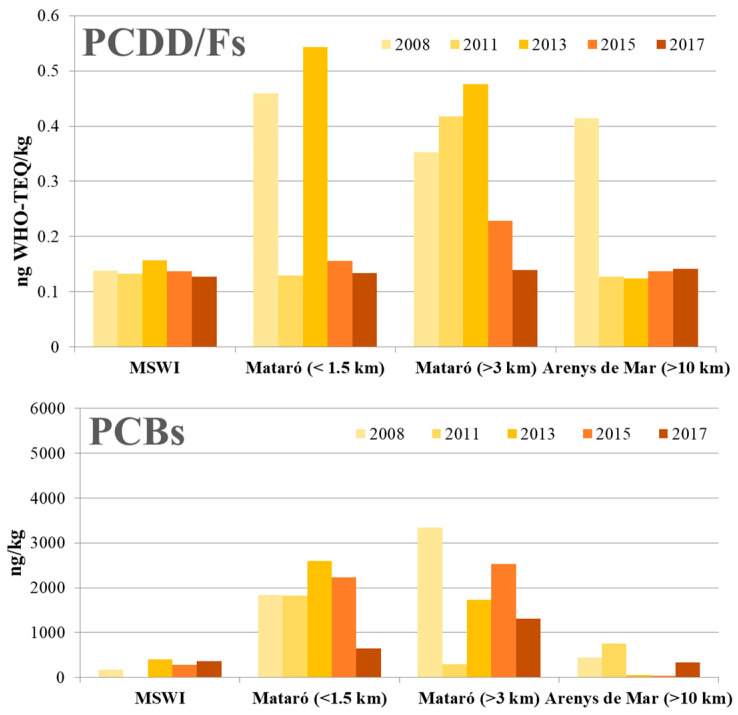
Temporal trend of the levels of PCDD/Fs and PCBs in samples of soils collected in the neighbourhoods of the MSWI of Mataró in 2008, 2011, 2013, 2015 and 2017.

In 2015, the mean concentration of PCDD/Fs in nine soil samples collected in different points and distances from the MSWI of Mataró was 0.23 ± 0.10 ng WHO-TEQ/kg, ranging from 0.14 to 0.42 ng WHO-TEQ/kg. No spatial differences were found, as the closest sampling sites (MSWI) showed very similar values to those found in the background location (0.15 and 0.14 ng WHO-TEQ/kg, respectively). Two years later, the average value of PCDD/Fs in the same 9 sampling sites was 0.13 ± 0.01 ng WHO-TEQ/kg, the levels being very similar in the different points (range: 0.12–0.15 ng WHO-TEQ/kg). Again, no differences were found when evaluating the spatial pattern of PCDD/Fs pollution, with the closest sampling sites (MSWI) presenting an even lower concentration than that found in the background site (Arenys de Mar): 0.13 vs. 0.14 ng WHO-kg. Interestingly, the soil concentrations of PCDD/Fs in Arenys de Mar did not change with time. The value obtained in 2017 meant a statistically significant decrease (*p* < 0.05) of 60% compared to that found in the background study, performed in 2008, when a mean level of 0.34 ng WHO-TEQ/kg was observed. On the other hand, the PCDD/Fs concentrations in soil found in the 2011 and 2015 campaigns were exactly the same (0.23 ng WHO-TEQ/kg). Therefore, a decrease between 2015 and 2017 was found, although it did not reach a level of statistical significance (*p* > 0.05). In both surveys (2015 and 2017), the highest chlorinated congeners (OCDD and 1,2,3,4,6,7,8-HpCDD) were the only compounds presenting detectable amounts in all the samples. Contrastingly, the two most toxic congeners (2,3,7,8-TCDD and 1,2,3,7,8-PeCDD) could not be detected in any sampling point. The temporal evolution of the total concentration of PCDD/Fs in soils around the facility is depicted in Figure 2.

In 2015, the total concentration of the sum of 7 environmental marker PCBs (28, 52, 101, 118, 153, 138, and 180) in the nine samples of soil was 1653 ± 1792 ng/kg, ranging from 39 ng/kg at the background (B) site to 5200 ng/kg at NE2 point. In 2017, the average level of PCBs decreased to 628 ± 461 ng/kg, with values ranging from 280 to 1700 ng/kg (I1 and NE2′ points, respectively). Moreover, a clear decrease was found not only in comparison to the most precedent survey (2015) but also with those previously conducted, with reductions of 130%, 41%, 167% and 163% with respect to 2008, 2011, 2013, and 2015, respectively. However, because of the large variability of results, the decrease was not statistically significant (*p* > 0.05). The levels of PCBs in the soils around the plant were quite similar to those found in Arenys de Mar (B and B’), considered as background site. The PCB congener profile in soil showed a predominance of heavier congeners (PCB-138, PCB-153, and PCB-180), while the most volatile PCBs presented the lowest concentrations. This fact would be closely related to the physicochemical characteristics of the different PCB congeners. High molecular weight PCBs are bound to the particle phase of emitted air, so they tend to deposit closer to emission sources and accumulate in soil, which acts as a sink for heavy weight PCB congeners [21].

**Figure 3:**
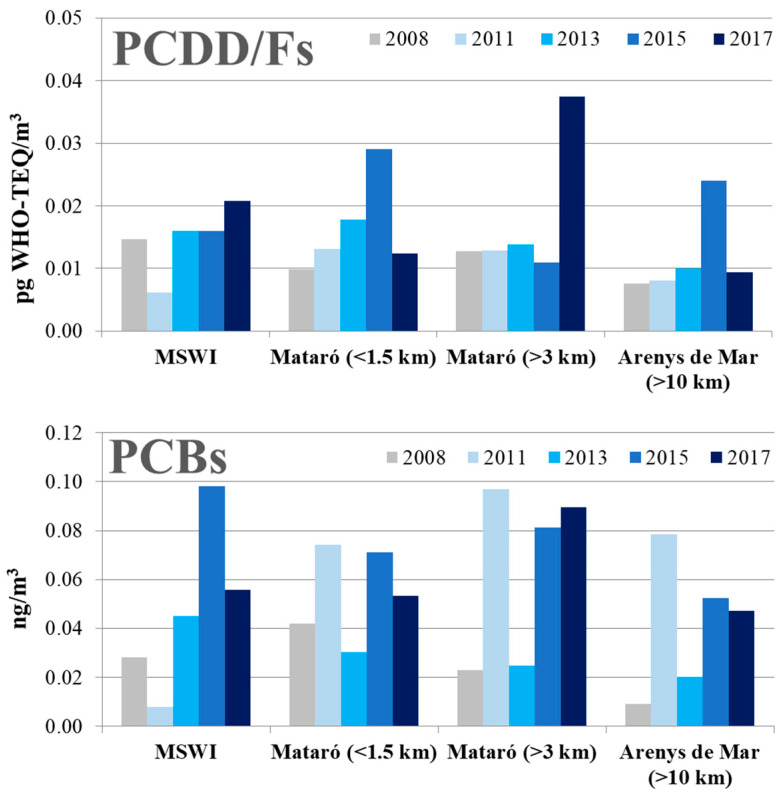
Temporal trends of the levels of PCDD/Fs and PCBs in air samples collected in the neighbourhoods of the MSWI of Mataró in 2008, 2011, 2013, 2015 and 2017.

The temporal trends of PCDD/Fs and PCBs in air samples collected between 2008 and 2017 in the surroundings of the MSWI of Mataró are depicted in Figure 3. In 2015, the airborne mean concentration of PCDD/Fs was 0.019 ± 0.008 pg WHO-TEQ/m^3^, with values ranging from 0.011 to 0.029 pg WHO-TEQ/m^3^. In the most recent survey (2017), the mean level of PCDD/Fs in air was very similar: 0.020 ± 0.013 pg WHO-TEQ/m^3^ (range: 0.009–0.037 pg OMS-TEQ/m^3^). This means a reduction percentage of 78%, 99% and 39% compared to the values obtained in the campaigns performed in 2008, 2011 and 2013, respectively. None of the air samples showed detectable amounts of 2,3,7,8-TCDD, in either 2015 or 2017. The PCDD/F congener profile showed a higher relative contribution of the heaviest compounds in the total concentration, with OCDD, 1,2,3,4,6,7,8-HpCDD and OCDF being the most relevant.

**Figure 4:**
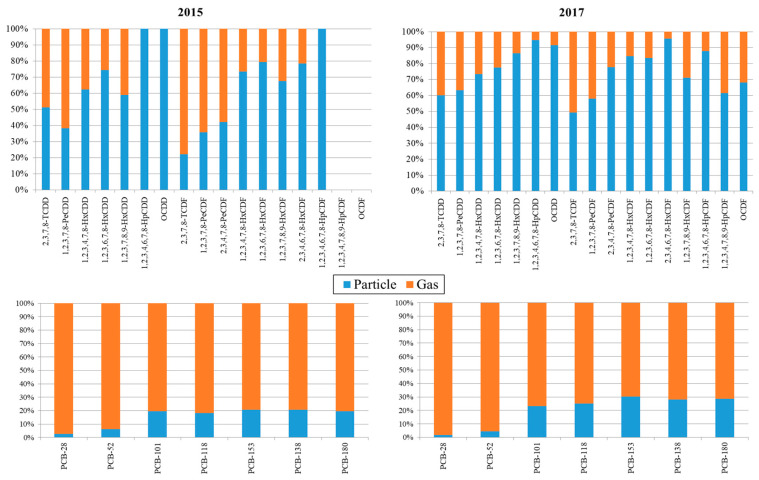
Congener profile of PCDD/Fs and PCBs in samples of air collected near the MSWI of Mataró in 2015 and 2017 according to the gas:particle distribution (ND: Not detected).

In 2015, the airborne mean concentration of PCBs was 0.075 ± 0.020 ng/m^3^, with minimum and maximum levels of 0.052 and 0.098 ng/m^3^, respectively. Two years later, the PCBs mean level in air was 0.061 ± 0.061 ng/m^3^, with values ranging from 0.047 to 0.089 ng/m^3^, respectively. This value means a statistically significant increase (*p* < 0.05) with respect to the levels obtained in 2008 (0.026 ng/m^3^) and 2013 (0.030 ng/m^3^). In contrast, there was a non-significant decrease compared to the 2011 study (0.064 ng/m^3^), and a significant decrease compared to the 2015 levels (0.076 ng/m^3^). Unlike soil, air samples showed a higher contribution of low molecular weight PCBs than heavier compounds. The congeners profiles of PCDD/Fs and PCBs in samples of air and soil collected in 2015 and 2017 are depicted in Figure 4.

**Figure 5:**
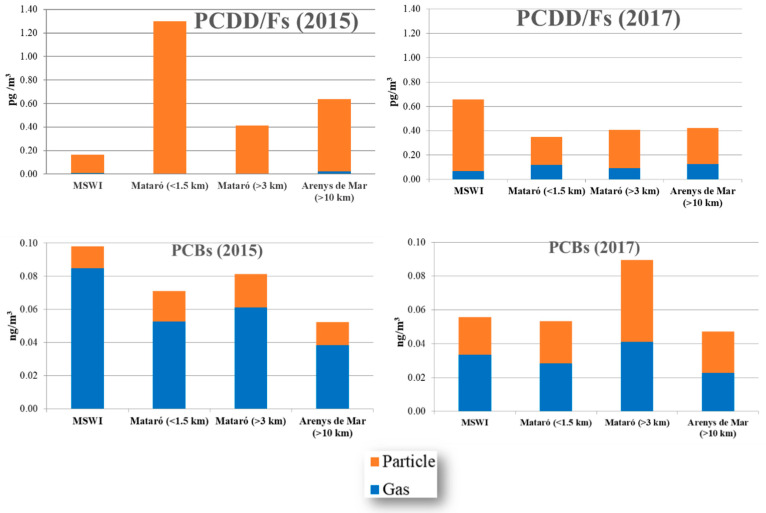
Gas/phase partitioning of PCDD/Fs and PCBs in air samples collected near the MSWI of Mataró in 2015 and 2017.

One of the novelties of the campaigns conducted in 2015 and 2017 is the specific study of gas/particle partitioning. Therefore, the concentrations of both, PCDD/Fs and PCBs, in the gas and particle phases were separately determined by analysing PUFs and microfiber filters, respectively. The contribution of the gas and the particulate fractions to the total amount of PCDD/Fs and PCBs is shown in Figure 5. In both campaigns, 2015 and 2017, PCDD/F congeners adsorbed to particles showed a higher contribution than PCDD/Fs in the gas phase, being especially evident in 2015. Contrastingly, a relatively higher contribution of PCBs congeners in the gas phase was noted, mainly in 2015. The three samples of air closer to the MSWI, including those collected in the city of Mataró, showed a slightly larger contribution of PCDD/Fs in the particle phase. However, the differences were minimal when comparing the pattern in the closest sites with that found in the background location. Regarding PCBs, higher contribution percentages of the gas phase were found in all the samples.

The environmental exposure to PCDD/Fs and PCBs through three different pathways (soil ingestion, dermal absorption, and air inhalation) was estimated, together with the total exposure. For that purpose, a previously validated health risk assessment methodology was applied [17]. The results of the environmental exposure to organochlorine compounds are summarized in Table 1. As frequently observed, air inhalation was the exposure route to PCDD/Fs with a higher contribution (87–97%), while the contribution of soil ingestion and dermal contact was minority.

The average total exposure to PCDD/Fs was 8.28·10^−6^ ng WHO-TEQ/kg·day in the area of direct influence of the MSWI emissions (considering the three closest points), while in Arenys de Mar, it was approximately 2.5 times lower (3.19·10^−6^ ng WHO-TEQ/kg·day). As for PCDD/Fs, air inhalation was the main exposure pathway to PCBs, with an average contribution of >60%. Furthermore, the total exposure to PCBs was similar near the facility than in the background site (1.85·10^−2^ vs. 1.60·10^−2^ ng/kg·day). In any case, the environmental exposure to PCDD/Fs and PCBs was not related to carcinogenic risk values above 10^−5^, which is considered as the threshold by the national legislation.

## 4. Discussion

From 2008 onwards, the environmental impact of the MSWI of Mataró, in terms of human health risks, has been periodically assessed. This monitoring program has been performed in parallel to the biological surveillance survey, which is aimed at the evaluating the body burdens of PCDD/Fs and PCBs in the facility workers and the population living nearby the plant. According to the most recent study, performed in 2012, the concentrations of PCDD/Fs in whole blood were very similar in the different population groups: 13.2, 13.1, 13.8, and 13.1 pg WHO-TEQ/g fat in workers, exposed people (living at <1 km in Mataró), unexposed people (living at >3 km in Mataró) and inhabitants of Arenys de Mar. This profile is the same as that of PCDD/Fs in soil samples collected in 2015 and 2017, since PCDD/F levels were very similar in the different sampling sites. It must be highlighted that soil is an accumulative matrix; therefore, soils are suitable long-term environmental monitors [22]. Furthermore, since 1995, when the biological surveillance study was initiated, PCDD/Fs in whole blood has not increased with time. In both exposed and non-exposed groups, PCDD/Fs concentrations experienced a slight increase from 1995 to 1999, followed by a slight decrease from 1999 to 2002 and stabilization afterwards, with no relevant differences between them [13]. The results of the environmental monitoring program, which was started in 2008, indicates higher PCDD/F concentrations in soils in the first surveys, being notably reduced with time. Regarding PCBs, whole blood concentrations of the sum the congeners 138, 153 and 180 showed fluctuations according to the population group. The inhabitants of Arenys de Mar (background) presented the highest body burdens of PCBs (0.34 µg/g fat), while those living in Mataró (>3 km) and the facility workers showed the minimum values (0.15 and 0.16 µg/g fat, respectively). PCBs in soils also showed larger fluctuations than PCDD/Fs, although PCB soil levels in Arenys de Mar were found to be relatively low compared to those found in the city of Mataró.

The concentrations of PCDD/Fs and PCBs in both environmental compartments, soil, and air, were also correlated with a number of meteorological parameters, including average, mean and maximum temperature, humidity, rainfall prior to and during the sampling, and wind speed. Data from the five campaigns (2008–2017) were considered. Very interestingly, the mean ambient temperature correlated positively with PCBs in air and negatively with PCDD/Fs in soil, especially when considering the evaluation period between 2008 and 2015 (R^2^ = 0.977 and R^2^ = 0.978 for air PCBs and soil PCDD/Fs, respectively). The temperature-dependence of airborne PCBs has been already suggested by other researchers [23,24]. Moreover, it has been also observed that air temperature decreases the magnitude of deposition processes and increases the revolatilization of PCDD/Fs [25].

Beyond the temporal and spatial analysis of the data, the current (2015 and 2017) concentrations of PCDD/Fs and PCBs in soil and air samples were compared with values from the scientific literature. In the last few decades, a number of environmental monitoring studies have been carried out near MSWIs in Catalonia (Spain). Vilavert et al. [26] found, in 2014, a mean PCDD/Fs soil concentration of 0.63 ng WHO-TEQ/kg near the MSWI of Tarragona. A decreasing temporal trend was also observed from the baseline study, performed in 1999, when the mean level of PCDD/Fs in soil was 1.20 ng I-TEQ/kg. More recently (in 2015 and 2016), it was observed that the mean PCDD/F concentrations in soils collected near the MSWI of Girona ranged 0.36–0.39 ng WHO-TEQ/kg [27]. Moreover, soil PCDD/Fs near the MSWI of Sant Adrià de Besòs were reported to be much higher; 3.60 ng WHO-TEQ/kg in 2014 [28], and between 9.0 and 22 ng WHO-TEQ/kg in 2018–2019 [29]. The soil levels found near the MSWI of Mataró are lower than those previously found in the surroundings of other Catalan MSWIs. Furthermore, they are also lower than values reported by a number of investigators around other incinerators around the world. In China, Zhou et al. [30] found that PCDD/Fs in soil samples collected around the largest MSWI in China ranged from 0.520 to 3.40 ng I-TEQ/kg, with an average of 1.49 ng I-TEQ/kg, while Deng et al. [31] reported a range of 0.362–8.55 ng WHO-TEQ/kg in soil samples collected near a MSWI in Shanghai.

Contrasting with soils, PCDD/Fs concentrations in air samples collected near the MSWI of Mataró (0.020 pg WHO-TEQ/m^3^ in 2017) were slightly higher than values from studies performed near the incinerators of Tarragona and Girona (0.010 and 0.011 pg WHO-TEQ/m^3^, respectively) [27,32]. In turn, they are much lower than levels found near the MSWI of Sant Adrià de Besòs, where airborne PCDD/Fs levels have ranged from 0.026 to 0.044 pg WHO-TEQ/m^3^ in recent years [28,33]. Moreover, they are also in the lower side of the range compared to values reported in Shanghai (China), where PCDD/F values ranged from 0.031 to 0.41 pg WHO-TEQ/m^3^ [31]. They are also lower than those found near a MSWI in northern China, where average values of PCDD/Fs in ambient air samples collected in 2016 and 2017 were 0.078 and 0.10 pg I-TEQ/m^3^, respectively [34].

Since the current legislation does not establish any emission limits for PCBs, data on the environmental burdens of PCBs near MSWIs are more limited. However, it must be noted that PCB levels in soils sampled in the surroundings of Mataró are quite low. Domínguez-Morueco et al. [35] found ten times higher concentrations of PCBs in soils collected near the most important petrochemical area in Spain, with values ranging from 6620 to 14,070 ng/kg in soils. Very high levels of PCBs were also found in soil samples from northern Vietnam, with concentration ranges of 14,500–130,000 ng/kg [36]. In turn, airborne PCBs levels in Mataró were similar to those previously reported in other industrial and urban areas worldwide [37,38].

In addition to the analysis of chlorinated compounds in air samples, a specific gas:phase partitioning study was conducted. Regarding PCDD/Fs, the 3 points located closest to the facility showed a higher contribution of PCDD/Fs in particles than in gas, especially in 2015. On the other hand, the two phases presented a very similar contribution at the reference point (Arenys de Mar). Hence, it can be concluded that the sampling sites near the MSWI are subjected to different PCDD/Fs emission sources, either industrial (including the own incineration plant) or urban (especially in the city of Mataró), than the background site. In turn, this differentiation could not be established for PCBs, which were more predominant in the gas phase, especially in the campaign of 2015. In agreement with data from the scientific literature [39], the contribution of the particulate phase was lower in the lighter congeners of PCDD/Fs, namely tetra- and penta-dioxins and furans (20–45%), while in the heavier congeners (i.e., hepta- and octa-dioxins and furans), the contribution of the particulate phase varied between 70% and 100%.

The profile of human exposure to polychlorinated compounds by the population living near the plant was compared with that resulting from the exposure to PCDD/Fs and PCBs in the background area (Arenys de Mar). Thus, the health risks associated to the environmental exposure to PCDD/Fs were 2.5 times higher near the facility, while those related to PCBs exposure were similar to those found in the background site. Anyhow, the current carcinogenic risk was lower than the current threshold used by the national legislation as a warning level [40]. In 2012, we estimated that the dietary exposure to PCDD/Fs by the Catalan population was 2.3·10^−4^ ng WHO-TEQ/kg·day [41]. This value is 2–3 orders of magnitude higher than the environmental exposure calculated from the PCDD/Fs concentrations in air and soil samples collected around the plant. More specifically, according to exposure calculations, diet would mean >94% of the total exposure to PCDD/Fs in the area of influence of MSWI. In a similar dietary study, the dietary intake of PCBs by the same Catalan population was estimated in 11.5 ng/kg·day [41]. Considering this dietary intake, food consumption would be the most contributing pathway to PCBs exposure, being 2–4 orders of magnitude higher than the environmental exposure. Therefore, dietary exposure would be greater than 99% of the total exposure. In agreement to data from the scientific literature [42,43], these estimations clearly indicate that food consumption is the most contributing factor to the total health risks derived from the exposure to PCDD/Fs and PCBs.

## 5. Conclusions

In the period 2008–2017, an environmental monitoring program was conducted around the MSWI of Mataró, as a support tool for the biological surveillance survey simultaneously performed. Soil PCDD/Fs concentrations showed a significant decrease compared to the values found in previous studies (2008 and 2013). In turn, airborne PCBs showed a significant increase with respect to the baseline (2008) survey. These changes could be related to differences of meteorological conditions among samples. The ambient temperature played a key role in each study, as mean temperature correlated positively with PCBs in air and negatively with PCDD/Fs in soil. However, no important spatial differences were noted for both, PCDD/Fs and PCBs, in air and soil, being the concentrations similar to those previously reported in the surroundings of other waste incinerators. The gas:phase partitioning study highlighted the potential influence of some industrial and/or urban emission sources of PCDD/Fs near the facility since the particle phase was predominant in the closest sampling sites. The MSWI could be actually one of these emission sources. Furthermore, the human exposure to PCDD/Fs was 2.5-fold higher compared to that from the background area. In any case, the environmental exposure to these chemical pollutants is still minor in comparison to their dietary intake. In addition, the current cancer risk values are also well below threshold values. The global assessment of the data regarding the different pollutants (PCDD/Fs and PCBs) and matrices (soil and air) indicates that there has not been a general increase in the environmental pollution around the facility.

## Figures and Tables

**Table 1:** Environmental exposure to PCDD/Fs (ng WHO-TEQ/kg·day) and PCBs (ng/kg·day) by the adult population living in different locations around the MSWI of Mataró.

		MSWI	Mataró (<1.5 km)	Mataró (>3 km)	Arenys de Mar (>10 km)
**PCDD/Fs**	Soil ingestion	1.98·10^−7^	2.06·10^−7^	2.19·10^−7^	2.19·10^−7^
	Dermal absorption	2.11·10^−7^	2.16·10^−7^	2.33·10^−7^	2.33·10^−7^
	Air inhalation	6.22·10^−6^	3.75·10^−6^	1.36·10^−5^	2.74·10^−6^
	**TOTAL**	**6.63·10^−6^**	**4.17·10^−6^**	**1.41·10^−5^**	**3.19·10^−6^**
**PCBs**	Soil ingestion	5.57·10^−4^	9.99·10^−4^	2.05·10^−3^	5.23·10^−4^
	Dermal absorption	2.77·10^−3^	4.97·10^−3^	1.02·10^−2^	2.60·10^−3^
	Air inhalation	1.52·10^−2^	1.45·10^−2^	2.44·10^−2^	1.29·10^−2^
	**TOTAL**	**1.85·10^−2^**	**2.05·10^−2^**	**3.67·10^−2^**	**1.60·10^−2^**
